# Retraction: Analysis of disease burden and future trends of ischemic heart disease in China and globally, 1990–2023

**DOI:** 10.1371/journal.pone.0351803

**Published:** 2026-06-17

**Authors:** The PLOS One Editors

After publication of this article [1], concerns were raised regarding the use of data from the Institute for Health Metrics and Evaluation (IHME)/Global Burden of Disease (GBD) 2023 dataset.

Specifically, the study utilised the GBD dataset prior to its public release, raising concerns that the version used may include incorrect or provisional data. The *PLOS One* Editors assessed Tables 1 and 2 in [1] based on the official GBD 2023 dataset released on October 25, 2025. This assessment identified discrepancies between the published tables and the official GBD 2023 dataset. These discrepancies primarily affect the age‑standardised rates, with smaller numerical differences also observed in absolute numbers. The first author stated that after they accessed the data, there was an update to the publication policy for the GBD dataset, such that publication of global-level data requires additional permissions; the first author requested withdrawal of the article [1] on the basis that its publication did not comply with the publication policy in place for the GBD dataset.

The *PLOS One* Editors have been unable to obtain further information from IHME about potential differences between the dataset used and the final released version of the GBD 2023 dataset, or about restrictions on data use and publication policy as applicable to this study. The concerns about the accuracy and completeness of the dataset used therefore could not be resolved. In light of the first author’s request and the unresolved concerns about adherence to data use requirements and about reliability of the published results, the *PLOS One* Editors retract this article.

ZZ did not respond to the final editorial decision. GY and LW either did not respond directly or could not be reached.
